# Nonconventional Techniques in Plant Alkaloid Extraction: A Decade of Progress (2014–2023)

**DOI:** 10.1002/cbdv.202403225

**Published:** 2025-04-03

**Authors:** Victor Menezes Sipoloni, Milena Costa Bassicheto, Maria Vitória de Oliveira, Ana Beatriz dos Santos de Souza, Gabriella Melo Alves, Thiago André Moura Veiga

**Affiliations:** ^1^ Programa de Pós‐Graduação em Biologia Química, Instituto de Ciências Ambientais Químicas e Farmacêuticas Universidade Federal de São Paulo Diadema Brazil; ^2^ Instituto de Ciências Ambientais, Químicas e Farmacêuticas Universidade Federal de São Paulo Diadema Brazil; ^3^ Departamento de Química, Instituto de Ciências Ambientais Químicas e Farmacêuticas, Universidade Federal de São Paulo Diadema Brazil

**Keywords:** alkaloids, green solvents, microwave extraction, plant species, supercritical fluid extraction

## Abstract

Plant metabolism encompasses primary and secondary pathways, with secondary metabolism yielding diverse natural products crucial for plant adaptation and ecosystem interactions. Among these products, plant‐derived alkaloids are bioactive compounds of significant pharmacological interest, traditionally extracted using conventional solvents known for their environmental and health hazards. Historically, alkaloid extraction relied on acid–base methods, which separate these compounds based on their solubility under different pH conditions. Since the 19th century, extraction techniques have evolved from traditional methods to modern approaches such as supercritical fluid extraction (SFE), microwave‐assisted extraction (MAE), ultrasound‐assisted extraction (UAE), and the use of ionic liquids (ILs) and deep eutectic solvents (DESs). These advanced methods optimize yield, reduce extraction times, and enhance sustainability by minimizing solvent use and environmental impact. This review explores the evolution of alkaloid extraction methodologies from 2014 to 2023, emphasizing eco‐friendly techniques. It discusses the principles and applications of SFE, MAE, UAE, ILs, and DESs in extracting alkaloids from plants, highlighting their pivotal role in modern natural product chemistry. These advancements underscore ongoing efforts to develop efficient and sustainable practices in alkaloid extraction, which is essential for pharmaceutical and medicinal applications.

## Graphical abstract

This figure seeks to represent the evolution of the different unconventional approaches that were adopted for the extraction of plant‐derived alkaloids during the decade from 2014 to 2023.

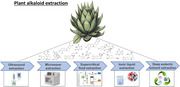

## 1 Introduction

Plant metabolism is divided into primary and secondary pathways. Primary metabolism includes essential processes like glycolysis and the Krebs cycle, which produce energy, reducing equivalents, and fundamental biomolecules (carbohydrates, proteins, lipids, and nucleic acids). In contrast, secondary metabolism involves compounds not directly linked to energy production or structural functions. These metabolites vary among plant species, contributing to their uniqueness [1].

Plant‐derived compounds play a crucial role in adapting of plants to their environments, enabling them to interact with diverse ecosystems effectively. These molecules, collectively known as natural products (NPs), enhance a species' survival by serving various biological functions. For instance, they can act as antibiotics, antifungals, and antivirals, protecting plants from pathogens. In addition, they may exhibit anti‐germination properties or toxic activities toward other plants, serving as phytoalexins. Moreover, certain metabolites function as UV protectants, absorbing ultraviolet light and thereby preventing damage to leaves [2]. Secondary metabolites are typically classified based on their biosynthetic pathways. The main families of these molecules include phenolic compounds, terpenes/steroids, and alkaloids.

Alkaloids exhibit remarkable structural diversity and are synthesized through various biosynthetic pathways, being present in about 20% of known vascular plants [3]. According to the *Dictionary of Natural Products* database (https://dnp.chemnetbase.com/chemical/ChemicalSearch.xhtml?dswid=‐733), over 49 600 natural alkaloids—encompassing those found in both fungi and plants—have been identified and cataloged. This vast diversity highlights the significance of alkaloids in nature and their potential applications in various fields, particularly in drug discovery and development [4].

These NPs are low molecular weight compounds, typically characterized by their alkaline nature due to the presence of a heterocyclic ring containing a nitrogen atom, which contributes to their pharmacological activity. The term “alkaloid” was first introduced by W. Meissner, an apothecary from Halle, in 1819. He noted that these compounds resembled alkalis in appearance and thus named them alkaloids [5]. Friedrich Sertürner, an apothecary's assistant from Westphalia, achieved the first isolation of morphine (**1**) Figure 1, a pivotal alkaloid, in 1805, marking a significant milestone in chemistry and pharmacology. Subsequently, pharmacists Pierre Joseph Pelletier and Joseph Benaimé Caventou utilized Sertürner's methodology to isolate a diverse array of alkaloids between 1817 and 1821. Among these were notable compounds such as brucine (**2**), febrifuge (**3**), quinine (**4**), caffeine (**5**), and veratrine (**6**) [6].

FIGURE 1Chemical structures of natural products isolated from plant material through diverse extraction techniques.

**Figure:**
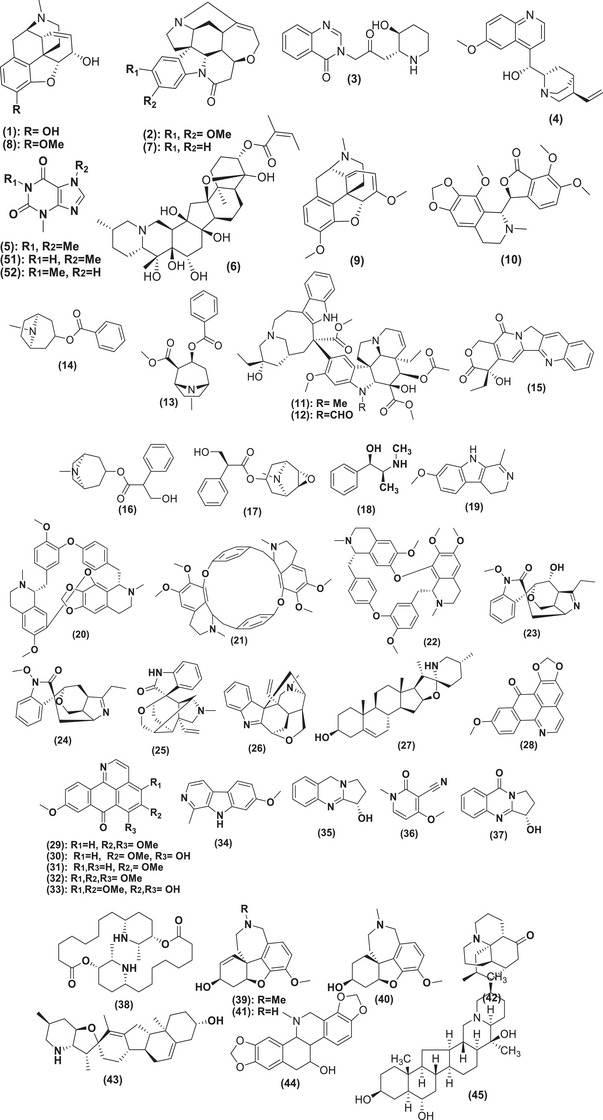

**Figure:**
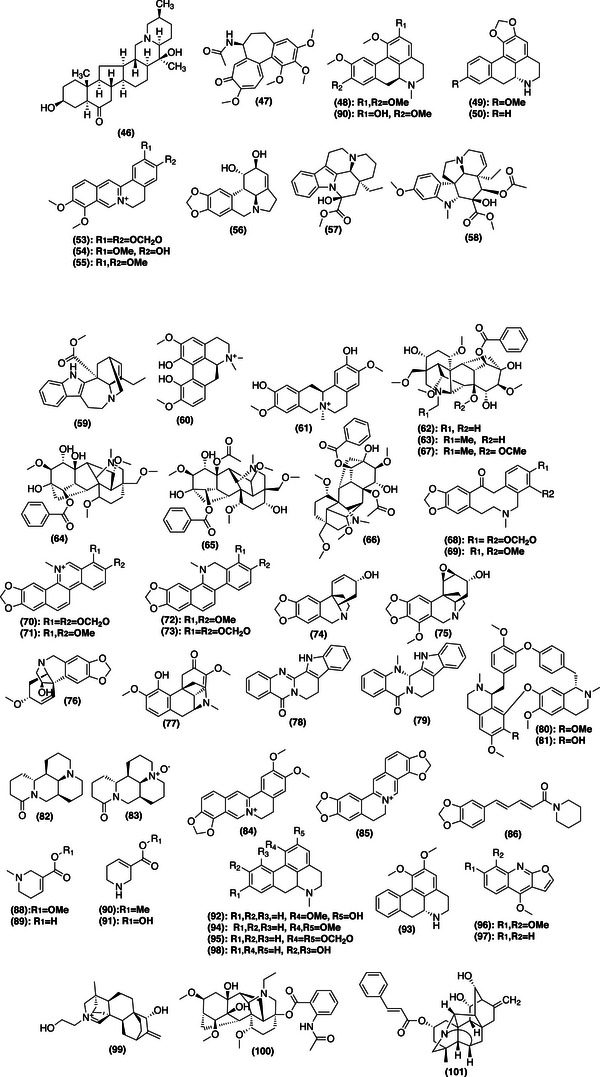

Alkaloids are classified into true alkaloids, pseudoalkaloids, or protoalkaloids based on their structural and biosynthetic features. True alkaloids are characterized by having nitrogen incorporated into a heterocyclic ring system, which originates from amino acid biosynthesis. Pseudoalkaloids, on the other hand, incorporate nitrogen from amino acids through transamination reactions. However, their carbon skeleton derives from pathways other than amino acid biosynthesis. Protoalkaloids originate from *N*‐methylation and decarboxylation reactions of amino acids. In these compounds, the nitrogen atom is not part of a heterocyclic system but is instead incorporated through methylation and subsequent decarboxylation processes. These classifications reflect the diverse biosynthetic pathways and structural characteristics that define alkaloids in nature [7, 8, 9, 10].

### 1.1 The Vital Role of Alkaloids in Pharmacology

Plant extracts containing alkaloids have historically been employed empirically as medicines, poisons, and “magic potions.” Dating back to 4000 bc, Sumerians used opium for its sedative and analgesic effects. Similarly, civilizations in the Americas—including the Incas, Aztecs, Mayans, Olmecs, and Toltecs—contributed to medicinal knowledge by using alkaloid‐containing substances such as (**4**), ipecacuanha, and coca for various therapeutic purposes [11].

Before 1800, most drugs were prepared as tinctures, decoctions, or infusions using water‐based or water/ethanol plant extracts. While some of these preparations were highly effective or toxic, their precise chemical compositions remained unknown. However, during the 19th century, advancements in organic chemistry and chemical analysis enabled the isolation and characterization of several compounds from plants [12]. Following the purification of (**1**), Pelletier and Caventou achieved notable milestones by isolating strychnine (**7**) from the seeds of Asian *Strychnos* trees in 1818. They also successfully extracted (**4**) from Cinchona in 1820 [13, 14].

In this scenario, the main plant families that biosynthesize alkaloids are Amaryllidaceae, Apocynaceae, Asteraceae, Berberidaceae, Boraginaceae, Elaeagnaceae, Erythroxylaceae, Fabaceae, Liliaceae, Loganiaceae, Menispermaceae, Papaveraceae, Ranunculaceae, Rubiaceae, Rutaceae, Solanaceae, and Zygophyllaceae [5]. However, alkaloids can also be found in other biological sources, indicating their widespread occurrence and significance in nature. The use of medicinal plants for treating various types of diseases has become increasingly popular in society. Since ancient times, plants have been used to ease pain and cure diseases long before the development of modern medicines [15].

Armand Séquin and Friedrich Sertürner were pioneers in the study and application of purified NPs [15], with one of their most important discoveries being morphine (**1**), an alkaloid known for its sedative effects and use in pain relief. Morphine is derived from opium, which comes from *Papaver somniferum* (the opium poppy) [15, 16, 17, 18]. Their work led to the identification and isolation of other alkaloids from opium, including codeine (**8**), thebaine (**9**), and narcotine (**10**), which contributed to the creation of the opioid drug class, recognized for its powerful analgesic properties [15].

Alkaloids also play an essential role in the treatment of diseases like cancer. For example, vinblastine (**11**) and vincristine (**12**), extracted from *Catharanthus roseus* (a member of the Apocynaceae family), are used in cancer therapies [19, 20]. Cocaine (**13**), derived from *Erythroxylum coca*, has been used as a local anesthetic since the 19th century [21], though its toxicity and addictive nature led to the search for safer alternatives, such as topacocaine (**14**), which offers similar effects with reduced toxicity.

In recent years, camptothecin (**15**), extracted from *Camptotheca acuminata*, has shown anti‐tumor properties by inhibiting topoisomerase I, a crucial enzyme in tumor cells [22]. In addition, *Atropa belladonna* yields atropine (**16**) and scopolamine (**17**), the latter being the main component of Buscopan, used for its analgesic and sedative properties in treating abdominal pain and cramps [23]. Ephedrine (**18**), extracted from *Ephedra*, is used for its sympathomimetic effects, though it can have adverse cardiovascular effects, and it is often used by athletes to enhance performance [24]. These historical examples highlight the significance of studying plant‐derived alkaloids due to their vast therapeutic potential. Despite some alkaloids' toxicity, their contributions to medical therapies are invaluable [25].

### 1.2 Fundamentals of Classical Alkaloid Extraction From Plants: A Comprehensive Overview

Extraction plays a crucial role in separating and recovering bioactive compounds from plant material, thereby transforming the matrix into a sample suitable for analysis. Traditional techniques such as decoction, maceration, infusion, digestion, and percolation, known since the 11th century, represent some of the oldest employed methods. In the 18th century, the introduction of the “Soxhlet extraction” technique integrated aspects of digestion and decoction methods [26, 27].

The limitations of traditional extraction methods prompted the development of numerous methods for extracting plant‐derived alkaloids [28]. Therefore, alkaloids are traditionally extracted from crude plant extracts using acid–base extraction methods. This method exploits the fact that alkaloids can exist in two forms: as ionic ammonium salts or as neutral free bases, depending on the pH conditions [29]. In plant extracts, these compounds are typically found as polar salts of organic acids, making them soluble in polar solvents such as methanol. Conversely, the free base forms of most alkaloids are insoluble in water but can be effectively extracted using nonpolar solvents such as benzene, dichloromethane, diethyl ether, chloroform, or ethyl acetate [30].

In recent decades, unconventional extraction methods, designed to be more environmentally friendly, have been introduced. These methods aim to reduce the use of synthetic and organic chemicals, decrease operating time, and improve both extract yield and quality. Examples include ultrasound‐assisted extraction (UAE), microwave‐assisted extraction (MAE), and supercritical fluid extraction (SFE) [31]. In addition, green solvents such as ionic liquids (ILs) and deep eutectic solvents (DESs) are being employed to replace traditional hazardous solvents widely used in industry [27].

**FIGURE 2:**
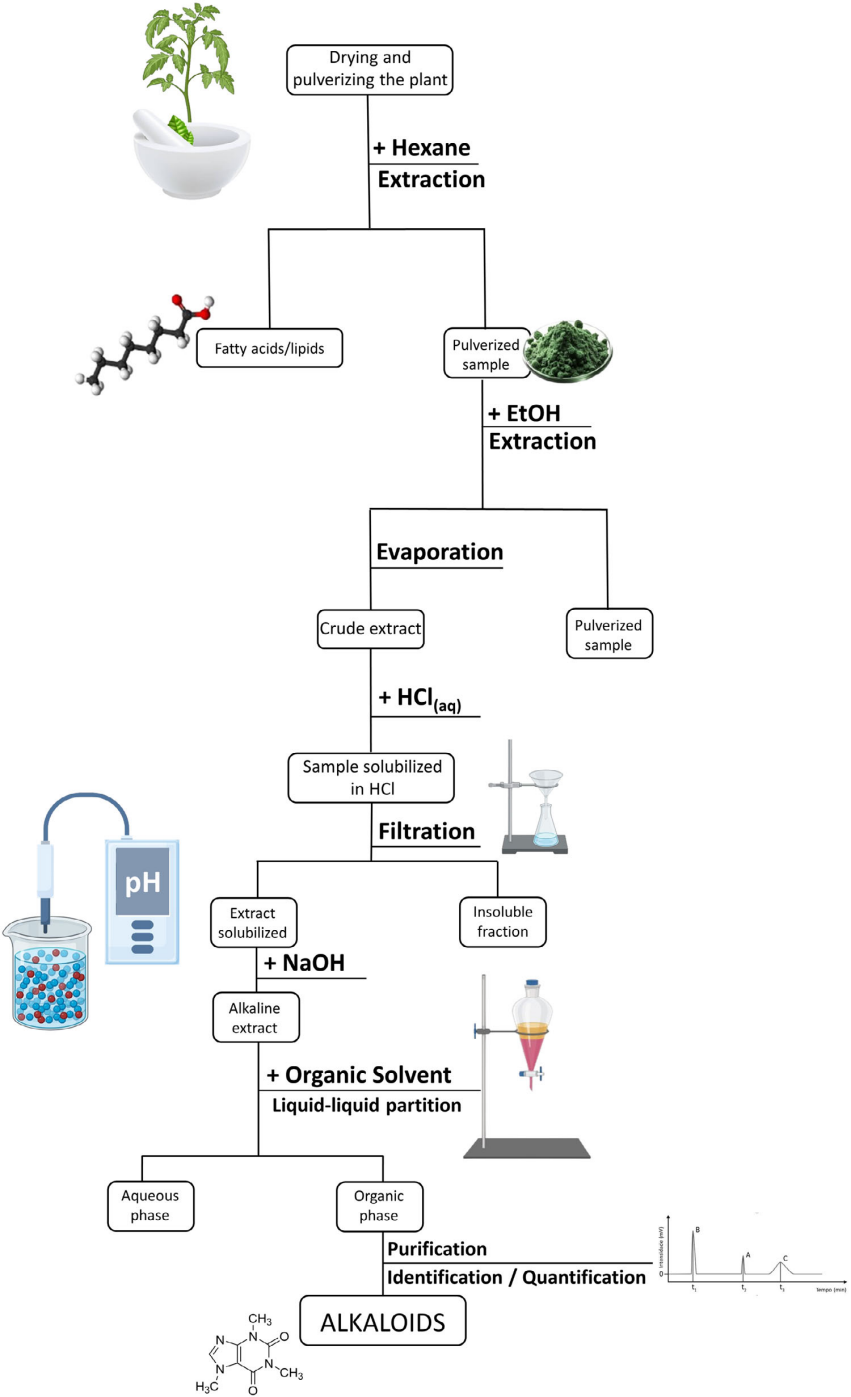
General procedure for extracting plant alkaloids. An acid–base extraction flowchart.

Acid–base extraction remains a core technique for isolating alkaloids from plants, which is crucial in both organic chemistry and pharmaceuticals [32]. This method, dating back to the early days of modern chemistry, stems from significant breakthroughs like morphine isolation [33], the extraction of quinine [34], and the discovery of harmaline (**19**) from *Peganum harmala* [35]. While efficient and versatile, the method has some drawbacks, such as the potential for compound degradation or artifact formation due to its multi‐step nature [36, 37]. The process typically involves using nonpolar solvents to extract lipids, followed by polar solvents to extract alkaloids, which are then acidified and alkalinized to convert them into their free base form (Figure 2). Despite its effectiveness, further advancements are needed to improve the efficiency of acid–base extraction [38, 39, 40]. Based on this context, this review offers a comprehensive exploration of plant metabolism, with a particular emphasis on the critical roles of secondary metabolites. It focuses on plant‐derived alkaloids, a key class of NPs, highlighting their pharmacological importance and their impact on medicine, from early isolations to modern therapeutic agents. In addition, it covers advancements in extraction methods, emphasizing the shift toward modern techniques from 2014 to 2023.

## 2 Materials and Methods

To track and investigate the most recent methodologies for extracting plant‐derived alkaloids from 2014 to 2023, an extensive literature survey was performed using the following databases: SciFinder^n^, Web of Science, Scopus, and PubMed. The selection of keywords aimed to refine and enrich our strategy, ensuring more objective and relevant results. The descriptors chosen for this survey are listed below, and the Boolean term “AND” was employed among the descriptors. The Rayyan platform (https://www.rayyan.ai/) efficiently assisted in the removal of duplicate data.

- “Microwave plant alkaloid extraction”
- “Supercritical fluid plant alkaloid extraction”
- “Ionic liquid plant alkaloid extraction”
- “Deep eutectic solvent plant alkaloid extraction.”

## 3 Results and Discussion

Modern extraction techniques, such as SFE, MAE, UAE, pressurized liquid extraction (PLE), and DESs, have revolutionized NP chemistry by enhancing efficiency and sustainability. These methods offer advantages over traditional techniques: SFE uses supercritical CO_2_ for high yields with minimal solvents, promoting green chemistry. MAE and UAE use energy waves to break plant cell walls, accelerating extraction and improving compound recovery. PLE employs high pressure and temperature to increase solvent penetration. DESs, biodegradable and nontoxic, effectively dissolve diverse NPs. Historically, SFE gained prominence in the 1980s, MAE in 1986, and UAE in the 20th century. ILs were explored in 1982, while DESs emerged in 2003, with natural deep eutectic solvents (NADESs) following in 2011 [41, 42, 43, 44, 45, 46, 47, 48, 49, 50, 51, 52, 53, 54, 55, 56, 57]. These innovations optimize phytochemical extraction while aligning with sustainability goals. The discussion will further explore their role in obtaining bioactive plant‐derived alkaloids.

### 3.1 Highlights and Trends of Recent Advancements in Extraction Techniques

Based on MAE strategies, initially, our survey identified a total of 201 articles across the searched databases. After applying exclusion criteria, we selected 12 articles that specifically detailed methodologies for obtaining alkaloids, primarily focusing on extracting bioactive alkaloids from plants. Most of these investigations aimed to enhance extraction efficiency through comparative methodologies, often employing microwave‐assisted or ultrasound‐assisted methods. These studies involved the extraction, isolation, and identification of 24 different alkaloids using MAE strategies. Some studies did not specify the identified alkaloids, while others focused on extracting multiple metabolites. Before conducting full‐scale extractions, optimization of extraction methods was performed using various parameters to identify the most efficient conditions.

In our database search on supercritical fluids, we consulted 17 articles that investigated methodologies using supercritical fluids to extract diverse classes of alkaloids. Most studies aimed to optimize methods and compare them with traditional techniques of alkaloid extraction. These studies identified and isolated 22 alkaloids, although specific identifications varied among publications, with some studies focusing on multiple alkaloid extractions. Optimization of extraction methods with varied parameters preceded the comprehensive extraction process to achieve optimal efficiency. For ILs extraction methods, we reviewed nine articles detailing methodologies for extracting different classes of alkaloids. Moreover, since 2017, we have observed a notable increase in the use of eutectic solvents for extracting plant‐derived alkaloids. A total of 21 studies have been published on this approach, covering the extraction of 44 alkaloids. Since the inception of using these solvents in alkaloid extraction, there has been at least one study involving DESs per year. Notably, although more prevalent overall, NADESs have demonstrated greater prominence since 2018.

**FIGURE 3:**
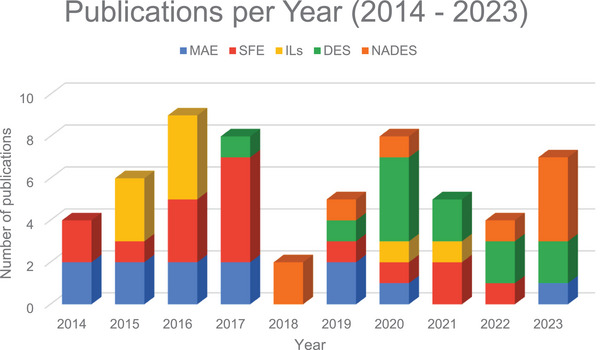
Annual analysis of publication trends in plant alkaloid extraction (2014–2023).

To provide a clearer overview of the annual publication trends, Figure 3 shows the number of publications per year related to the various extraction methods discussed in this review. As the graph illustrates, in 2014, the only topics with publications were MAE and SFE. From 2015 onward, research interests diversified, with new topics like ILs being explored. In 2018, the focus shifted as NADES emerged, while other topics saw a decline in activity. Beginning in 2020, DES started gaining more attention, with NADES becoming particularly prominent in 2023.

Over the years, publications on MAE have remained relatively steady, showing only slight fluctuations. Although there was a minor decline in 2021 and 2022, this trend reversed in 2023. SFE saw a gradual increase in interest, peaking in 2017, but the number of related publications declined afterward. ILs were a popular topic between 2015 and 2016, but interest dropped sharply in subsequent years, with only one publication in both 2020 and 2021. DES reached a peak in 2020, reflecting a growing interest during that period, but experienced a slight decline thereafter. This decline could be attributed to the significant growth in publications on NADES in 2023, which suggests a potential shift in focus and an increase in relevance or interest in this topic.

Overall, these trends reflect the dynamic nature of the field, with research interests evolving over time. New methodologies and solvents are continuously being explored and evaluated for their efficiency and applicability in extracting valuable plant‐derived alkaloids. The shift in focus suggests that while some topics gain prominence, others decline, highlighting the ongoing search for more effective and sustainable extraction methods.

### 3.2 Alkaloids Extraction Assisted by Microwave (MAE) and Ultrasound (UAE)

**FIGURE 4:**
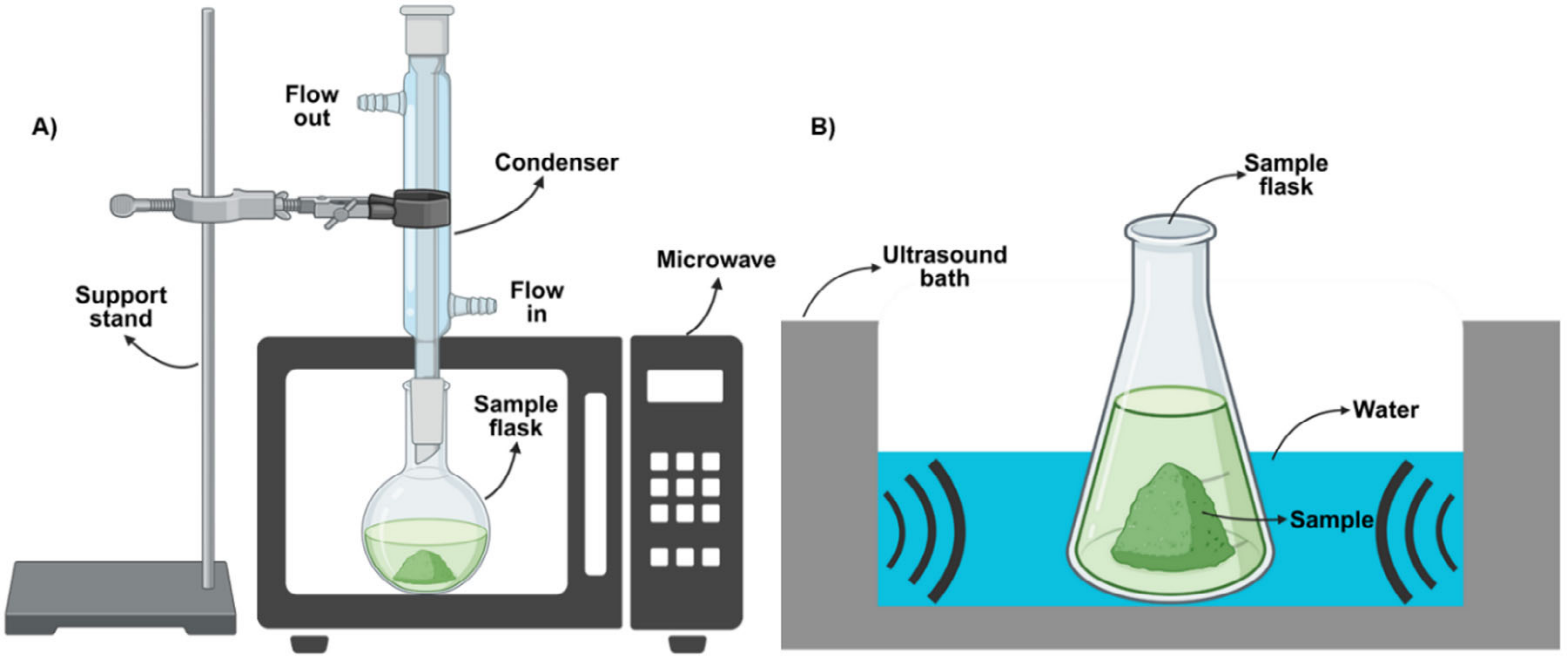
Illustration of a schematic representation of MAE and UAE method process.

In the MAE method, electromagnetic radiation is utilized to enhance extraction kinetics and improve extract quality. Microwave energy, absorbed by polar compounds based on their dielectric constant, dissipates as heat, enabling targeted heating of specific metabolites in plant samples for more efficient extraction. Traditional methods such as solvent extraction, leaching, and hydrodistillation are often deemed inefficient and time‐consuming due to the degradation of active metabolites. Effective pre‐extraction and extraction procedures are critical for processing bioactive constituents from plants [58, 59]. The food industry is actively seeking new extraction techniques to address safety concerns, environmental regulations, and the reduction of waste and energy consumption [60]. Modern methods like MAE and UAE have proven to be more efficient, cost‐effective, and faster compared to conventional solvent extraction methods. Emerging technologies such as UAE, microwave‐assisted techniques, and PLE offer sustainable and “green” solutions, yielding high‐purity extracts while reducing solvent and energy usage. In addition, UAE simplifies handling and processing, enhances product purity, and reduces solvent and energy requirements by operating at lower temperatures or eliminating the need for solvents [61, 62]. A schematic illustration of MAE (a) and UAE (b) methods is shown in Figure 4.

Based on the studies by Desgrouas et al. [63] and Liu al. [64], both publications focused on optimizing the extraction of bisbenzylisoquinoline alkaloids from *Stephania* species using MAE strategies. Several parameters, such as power levels, solvents, solid–liquid ratios, temperatures, and extraction times to enhance the extraction of cepharanthine (**20**) from *Stephania rotunda* Lour. [63]. The authors identified ethanol–water (50:50, v/v) with a solid–liquid ratio of 1/20 (w/v), operated at 100 W for 15 min at 80°C, as the optimal conditions, achieving high efficiency and reducing extraction time compared to the UAE (Table 1). In contrast, focusing on the extraction and purification of the alkaloids (**20**), cycleanine (**21**), and isotetrandrine (**22**) from *Stephania cepharantha*, a MAE combined with solid‐phase extraction (SPE) (MAE‐SPE) approach was employed. The optimized method involved heating plant material with 0.01 mol/L HCl at 100 W and 60°C for 2 min (Table 1), followed by purification using a polymeric cation exchange‐SPE (PCX‐SPE) cartridge, providing specific yields for each compound, demonstrating the efficacy of the approach in extracting and purifying alkaloids from the species [64]. Both studies underscore the effectiveness of MAE in enhancing extraction efficiency while reducing environmental impact compared to traditional methods.

**TABLE 1:** Summary of study conditions for alkaloid extraction using microwave‐assisted extraction (MAE) and ultrasound‐assisted extraction (UAE) techniques.

Target alkaloid(s)	Plant source	Technique	Optimized conditions	References
Cepharanthine (**20**)	*Stephania rotunda* Lour.	MAE	Ethanol–water (50:50, v/v); solid–liquid ratio 1:20 (w/v); 100 W; 15 min; 80°C	Desgrouas et al. [63]
Cycleanine (**21**), isotetrandrine (**22**), cepharanthine (**20**)	*Stephania cepharantha*	MAE‐SPE	0.01 mol/L HCl; 100 W; 2 min; 60°C	Liu et al. [64]
Humantenidine (**23**), humantenmine (**24**), gelsemine (**25**), koumine (**26**)	*Gelsemium elegans*	UAE/MAE	Methanol; 3–10 min, 1–3 extraction cycles; 40°C–52°C	Li et al. [65]
Diverse alkaloids	*Sophora flavescens*	MAATPE	Ethanol/ammonium sulfate; 780 W; 5 min; 90°C	Zhang et al. [66]
Solasodine (**27**)	*Solanum nigrum* L.	MAATEPE	Ethanol, ammonium sulfate, water (28:16:56, w/w/w)	Sakti et al. [67]
Bianfugedine (**28**), menisporphine (**29**), 6‐*O*‐demethylmenisporphine (**30**), bainfugecine (**31**), dauriporphine (**32**), dauriporphinoline (**33**)	*Menispermum dauricum*	MAE	70% ethanol; liquid–solid ratio 20 mL/g; 11 min; 60°C	Wei et al. [68]
Harmine (**34**), harmaline (**19**), vasicine (**35**)	*Peganum harmala*	MAE	80% ethanol (v/v); 0.2 g plant material; 600 W; 8 min; 80°C	Shang et al. [69]
Camptothecin (**15**)	*Nothapodytes nimmoniana*	MAE	Methanol; solid–liquid ratio1:50 g/mL; 150 W; 40°C	Patil et al. [70]
Betacyanins	*Gomphrena globose*	MAE/UAE	Ethanol; solid‐to‐liquid ratio 5 g/L; 8 min; 60°C	Roriz et al. [71]
Ricinine (**36**)	*Ricinus communis*	MAE	10% ethyl acetate in methanol; liquid–solid ratio 25 mL/g; 1000 W; 15 min; 175°C	Nebo et al. [72]
Vasicine (**35**), vasicinone (**37**)	*Justicia adhatoda*	MAE	Methanol; 1 g dried sample in 40 mL solvent; 1200 W; 6 min	Padhiari et al. [73]
Carpaine (**38**)	*Carica papaya*	MAE	80% aqueous ethanol; 1 kg powdered leaves; 800 W; 20 min; 60°C	Mandour et al. [74]

Li et al. [65] explored UAE/MAE methods to extract four indole alkaloids from *Gelsemium elegans*: humantenidine (**23**), humantenmine (**24**), gelsemine (**25**), and koumine (**26**). The optimized extraction parameters included solvent type, extraction time, temperature, liquid‐to‐solid ratio, and number of extraction cycles. Methanol was found to be the most effective solvent, yielding the highest concentrations of all alkaloids compared to acetonitrile, ethanol, and ethyl acetate. The study showed that increasing the extraction temperature from 40°C to 52°C, extending extraction time from 3 to 10 min, and performing 1–3 extraction cycles led to significant improvements in alkaloid yields under the optimized conditions [65].

Zhang et al. [66] introduced microwave‐assisted aqueous two‐phase extraction (MAATPE) as a novel method to extract alkaloids from *Sophora flavescens*. They combined MAE with aqueous two‐phase extraction (ATPE), using ethanol/ammonium sulfate as the solvent. Operating at 90°C and 780 W for 5 min, this approach yielded 63.78 ± 0.45 mg/g of alkaloids with a recovery rate of 92.09 ± 0.14%, showing significant improvement in yield and purity compared to traditional methods [66].

Building on this, Sakti et al. [67] also applied MAATPE to extract solasodine (**27**) from *Solanum nigrum* L. They optimized their method using ethanol, ammonium sulfate, and water (28:16:56, w/w/w) as the aqueous two‐phase system (ATPS), determined through response surface methodology (RSM). Their results demonstrated higher extraction yields and reduced extraction times compared to conventional approaches, highlighting the efficiency of MAATPE in extracting bioactive metabolites [67].

In addition, a method for the simultaneous quantification of six oxoisoaporphine alkaloids extracted from *Menispermum dauricum*: bianfugedine (**28**), menisporphine (**29**), 6‐*O*‐demethylmenisporphine (**30**), bainfugecine (**31**), dauriporphine (**32**), and dauriporphinoline (**33**). Optimal extraction conditions, determined through orthogonal experiments, involved using 70% ethanol as the solvent at 60°C for 11 min, with a solvent/solid ratio of 20 mL/g. This resulted in a total alkaloid content yield of 232.8 µg/g [68].

Three bioactive alkaloids—harmaline (**19**), harmine (**34**), and vasicine (**35**)—were simultaneously extracted and determined from *P. harmala* seeds using an efficient method. The extraction involved using 0.2 g of plant material with 6 mL of 80% ethanol (v/v) at 600 W and 80°C for 8 min. This process yielded 38.7 mg/g of (**19**), 17.3 mg/g of (**34**), and 5.9 mg/g of (**35**) [69].

The extraction of (**15**) from *Nothapodytes nimmoniana* was also investigated [70]. Solvents such as acetone, chloroform, ethanol, and methanol were used to optimize extraction conditions, including a solid‐to‐liquid ratio of 1:50 g/mL, 150 W microwave power, 40°C temperature, and particle size range of 0.84–0.42 mm. Methanol provided the highest extraction efficiency, yielding 3.349 ± 0.007 mg/g due to its favorable dielectric constant, viscosity, and polarity index. In conclusion, the extraction time was minimized to 2 min, and kinetic studies emphasized the substantial influence of stirring, power level, and temperature on extraction efficiency.

Roriz et al. [71] optimized the extraction of betacyanins from *Gomphrena globosa* using RSM with a combination of MAE and UAE. The optimal conditions—8 min extraction time, 60°C, 0% ethanol content, and a solid‐to‐liquid ratio of 5 g/L—yielded 39.6 ± 1.8 mg/g of betacyanins [71]. Another study was performed to extract ricinine (**36**) from *Ricinus communis* leaves. The optimal conditions—175°C, 1000 W, 15 min, 10% ethyl acetate in methanol, and a liquid‐to‐solid ratio of 25 mL/g—yielded approximately 1.5 mg/g of (**36**) per gram of dried leaves in 15 min [72].

In 2020, Padhiari et al. optimized a method to simultaneously quantify (**35**) and vasicinone (**37**) from different parts of *Justicia adhatoda* using MAE with methanol. They irradiated a 1 g dried sample in 40 mL of solvent at 1200 W for 6 min. This method outperformed maceration, ultrasonic‐assisted extraction, and heat reflux extraction in efficiency. Yields of (**35**) and (**37**) were highest in the leaf (6.39 and 3.60 mg/g, respectively), followed by the stem (4.00 and 1.89 mg/g) and the root (1.23 and 0.73 mg/g) [73]. A similar approach was used to enhance the extraction of carpaine (**38**) from *Carica papaya* leaves, resulting in an extraction efficiency of 18.75%. (**38**) was subsequently isolated as pure crystals from the leaf extracts, showcasing MAE's ability to efficiently extract and purify specific compounds from plants [74].

MAE and UAE offer distinct advantages and considerations in extracting bioactive compounds, particularly plant‐derived alkaloids, MAE utilizes electromagnetic radiation to selectively “fish” compounds, speeding up extraction kinetics and enhancing yield and purity. This method reduces solvent use and energy consumption. However, MAE requires precise optimization of parameters like power levels and extraction time to avoid heat‐induced degradation of sensitive compounds. Conversely, UAE utilizes sound waves to disrupt plant cell structures, facilitating the release of intracellular compounds with minimal energy and time. Operating at lower temperatures, UAE preserves thermally sensitive compounds and ensures a safer process. Yet, it may necessitate longer extraction times and careful control of sonication to prevent sample degradation or emulsification. Both techniques significantly improve extraction efficiency and purity, advancing applications in pharmaceuticals, food, and cosmetics industries. Their sustainable and effective extraction capabilities make MAE and UAE valuable tools in modern extraction methodologies.

### 3.3 Alkaloids Extraction by Supercritical Fluid

SFE, particularly with CO_2_ as the solvent, offers significant advantages in the extraction of alkaloids. It utilizes CO_2_ above its critical temperature and pressure to achieve high selectivity and efficiency in extracting target compounds. SFE minimizes the use of organic solvents, making it suitable for pharmaceutical and research applications. However, SFE requires precise optimization of parameters such as temperature, pressure, and cosolvent use to achieve good yields and maintain compound stability. Compared to traditional methods like maceration and UAE, SFE often yields lower quantities but produces more purified extracts, thereby streamlining downstream purification processes.

**FIGURE 5:**
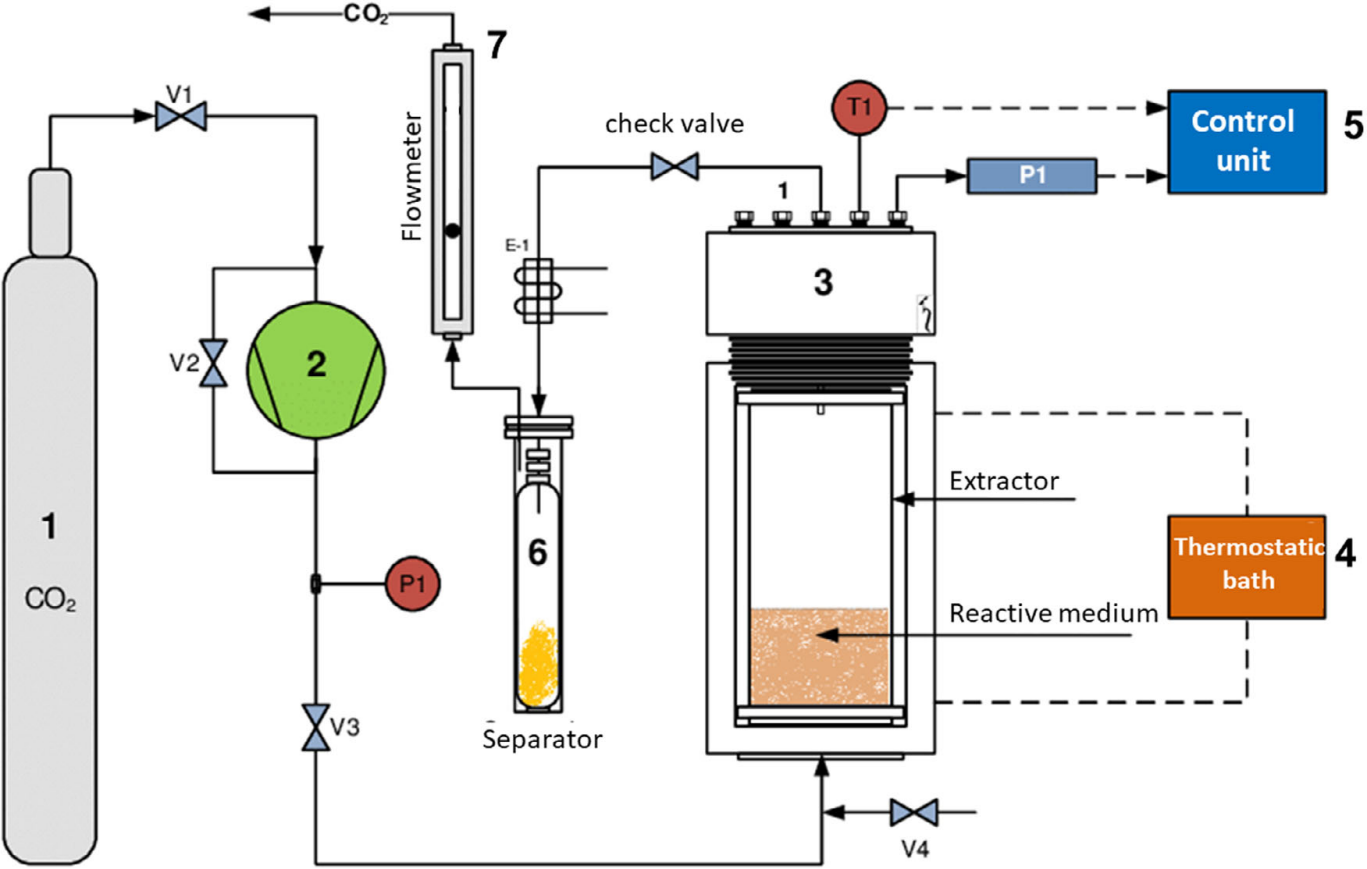
Diagram of an extraction unit by supercritical fluid. (1) CO_2_ cylinder; (2) CO_2_ pump; (3) extractor; (4) thermostatic bath; (5) control unit; (6) separator; (7) flowmeter.

Several studies (Table 2) illustrate the application of SFE (scCO_2_) and other advanced techniques in extracting specific alkaloids from natural sources, showcasing their potential for efficient extraction and yield optimization in pharmaceutical and research applications. A schematic illustration of an extraction unit is shown in Figure 5.

**TABLE 2:** Summary of study conditions for alkaloid extraction using supercritical fluid extraction.

Target alkaloid(s)	Plant source	Optimized conditions	References
Galanthamine (**39**)	*Narcissus pseudonarcissus*	25% (v/v) NH_4_OH; 70°C; 220 bar; 3 h	Rachmaniah et al. [75]
Lycopodine (**42**)	*Lycopodium clavatum*	300 bar; 40°C	Da Silva et al. [76]
Cyclopamine (**43**)	*Veratrum californicum*	SFE using copper coil setup yielded 0.66 mg/g	Turner et al. [77]
Chelidonine (**44**)	*Chelidonium majus*	Temperature, solvent density, and basified cosolvents varied; SFE followed by ESE	Gañán et al. [78]
Betacyanins	*Hylocereus polyrhizus*	scCO_2_ with 10% of cosolvents; 50°C; 25 MPa	Fathordoobady et al. [79]
Pentacyclic alkaloids	*Uncaria tomentosa*	Hydroalcoholic extract; 30 MPa; 45°C Pure scCO_2_; 10–45 MPa; 45°C	Calvo et al. [80]
Diverse alkaloids	*Melocactus zehntneri*	300 bar; 35°C	Brandão et al. [81]
Diverse alkaloids	*Fritillaria thunbergii*	90.3% ethanol; 61.3°C; 30.6 MPa; 2.9 h	Ruan et al. [82]
Colchicine (**47**)	*Gloriosa superba*	Liquid CO_2_ with 3% water as cosolvent; 60°C; 400 bar; 2 h	Balkrishna et al. [83]
Aporphine alkaloids	*Annona cherimola*	100 bar; 75°C; 15% methanol as cosolvent	Galarce‐Bustos et al. [84]
Diverse alkaloids	*Papaver bracteatum*	351 atm pressure; 90 mL modifier volume (9% v/w); 40°C; 15 min static, 55 min dynamic extraction	Salehi et al. [85]
Methylxanthines	Yerba mate leaves	Pure scCO_2_; 333 K; 26 MPa; 4.5 h Ethanol; 333 K; 26 MPa; 4.5 h 3. EtOH:H_2_O (7:3 m/m); 333 K; 26 MPa; 4.5 h	Hegel et al. [86]
Diverse alkaloids	*Sophora moorcroftiana*	31 MPa pressure; 70°C; 2.7 h	Hu et al. [87]

Rachmaniah et al. investigated the extraction of galanthamine (**39**) from *Narcissus pseudonarcissus* bulbs [75], focusing on optimizing extraction conditions such as particle size, CO_2_ density (temperature and pressure), flow rate, and plant material treatment. Ten alkaloids were identified in the scCO_2_ extracts, including (**39**), lycoramine (**40**), and norgalanthamine (**41**). The highest (**39**) yield (303 µg/g) was achieved at 70°C and 220 bar over a 3‐h period, using plant material with a particle size of 53–1000 µm moistened with 25% (v/v) of aqueous ammonia. Utilizing RSM, another study extracted lycopodine (**42**) from *Lycopodium clavatum* [76]. Optimal conditions were 300 bar and 40°C without pretreatment, resulting in 20.29% of (**42**). From *Veratrum californicum*, Turner et al. investigated various extraction techniques for obtaining cyclopamine (**43**) [77]. Eight methods were compared, including Soxhlet reflux, ethanol and benzene soaks, SFE, and MAE. The supercritical fluid method, using a copper coil setup, yielded 0.66 mg/g of (**43**), comparable to other methods but lower than that achieved with ethanol soak (8.03 mg/g).

Gañán et al. investigated both SFE and enhanced solvent extraction (ESE) methods for fractionating extracts from *Chelidonium majus* aerial and terrestrial parts [78]. They studied the effects of temperature, solvent density, and basified cosolvents on extraction yield, kinetics, and composition, comparing these methods with traditional approaches like Soxhlet and low‐pressure solvent extraction. Their findings highlighted SFE's initial selectivity for chelidonine (**44**) rich extracts, followed by an ESE step to enhance overall yields, albeit with a dilution of alkaloid concentration. Fathordoobady et al. assessed the feasibility of SFE for extracting betacyanin pigments from *Hylocereus polyrhizus*, comparing it with conventional solvent extraction methods [79]. The SFE strategy achieved comparable betacyanin contents and extraction efficiencies while significantly reducing organic solvent usage, suggesting environmental benefits over traditional methods. The application of SFE using CO_2_ and ethanol as an entrainer to extract *Uncaria tomentosa* bark to obtain pentacyclic alkaloids with reduced tannin and antioxidant levels. The study emphasized SFE's capability to selectively extract alkaloids when combined with ethanol, although it required substantial amounts of plant material for effective high‐pressure extraction [80].

Brandão et al. extracted alkaloids from the aerial parts of *Melocactus zehntneri* using supercritical CO_2_ [81]. They compared the global yield and alkaloid profile of SFE extracts with those obtained through conventional techniques, including maceration and UAE. SFE was conducted on both adult and young plant specimens in parallel experiments. The highest global yield was 0.68%, achieved at 300 bar and 35°C using adult plant material, followed by 0.52% from young material at the same temperature. For conventional methods, maceration showed the highest efficiency with a 14 ± 1% yield after 72 h, and slightly lower yields for 24 and 48 h (11 ± 2% and 12 ± 1%, respectively). UAE yielded 13 ± 1%, similar to maceration, indicating no additional benefit from ultrasonic waves for this material. Although SFE had a lower yield, it was the most selective for extracting alkaloids and also produced polysaccharide‐free extracts. This reduced the number of purification steps, making SFE a more sustainable technique due to the unique properties of scCO_2_. Similarly, Ruan et al. optimized SFE conditions for extracting alkaloids from *Fritillaria thunbergii* flowers, achieving high yields of total alkaloids, peimine (**45**), and peiminine (**46**). These studies demonstrate SFE's efficacy and selectivity, emphasizing its potential for producing high‐quality extracts with fewer purification steps and greater environmental benefits [82].

Recent studies have continued to demonstrate the effectiveness of SFE [83]. Colchicine (**47**) was successfully extracted from *Gloriosa superba* seeds. The researchers processed 3 kg of seed powder containing 0.70% (**47**), using liquid CO_2_ with 3% water as the cosolvent. Conducted at 60°C for 2 h, with pressures ranging from 200 to 450 bar, the optimal yield of 93.6% was achieved at 400 bar, corresponding to 27% of (**47**). Galarce‐Bustos et al. optimized SFE to extract alkaloids from *Annona cherimola*, evaluating the extracts' bioactivity profile [84]. Several aporphine alkaloids were identified, including *S*‐(+)‐glaucine (**48**), xylopine (**49**), and anonaine (**50**), and the optimal conditions are described in Table 2.

For the extraction of alkaloids from the organs of *Papaver bracteatum* L., Salehi et al. conducted an experimental design including a 2^(^ *^n^* ^− 1)^ fractional factorial and central composite design (CCD) to evaluate and determine optimal conditions: 351 atm pressure, 90 mL modifier volume (9% v/w), 40°C oven temperature, and extraction times of 15 min static and 55 min dynamic. This study highlights SFE's effectiveness in extracting specific alkaloids under optimized parameters [85].

The extraction of (**5**), theobromine (**51**), and theophylline (**52**) was also explored from dry yerba mate leaves [86]. The effects of cosolvents in both static and continuous loading methods were considered. Pure scCO_2_ demonstrated high selectivity for alkaloids but resulted in a low extraction yield of 0.39 g/kg of yerba mate. The addition of ethanol significantly increased the alkaloid content, reaching up to 5.0 g/kg with continuous loading. Using hydrated ethanol further enhanced the extraction yields, achieving 9.4 g/kg of alkaloids with continuous loading. The total amount of alkaloids was extracted from *Sophora moorcroftiana*, with the aim of mitigating issues associated with organic solvent residue and complex industrial production found in other extraction methods. Thus, parameters were systematically varied, including pressure, temperature, time, alkalization time, carrier ethanol concentration, and sample crushing time. Box–Behnken response surface analysis identified the optimal conditions (Table 2), resulting in an extraction yield of 68.88 µg of total alkaloids per gram of *S. moorcroftiana* [87].

### 3.4 IL Plant Alkaloid Extraction

ILs are a class of solvents known for their unique properties that exceed those classified as traditional. They possess negligible vapor pressure, are nonflammable, exhibit high thermal stability, and show low chemical reactivity, enhancing safety and environmental friendliness. Comprising organic cations paired with inorganic or organic anions, ILs remain liquid at room temperature, offering exceptional dissolving and extraction capabilities alongside a wide liquidus range. Their applications span synthesis, catalysis, electrochemistry, and analytical chemistry, demonstrating superior efficiency in extraction processes and reducing solvent usage while yielding higher outputs [88, 89].

**FIGURE 6:**
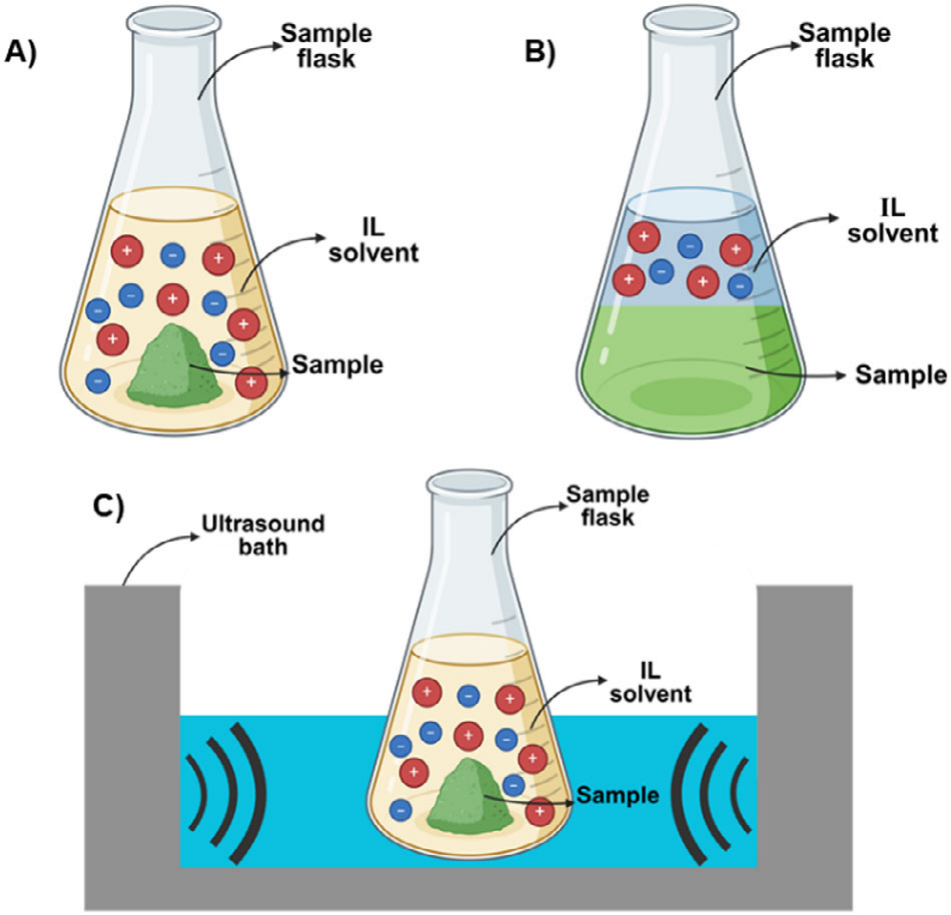
Different methods types for extraction with IL. (A) Aqueous IL solution; (B) aqueous two‐phase systems; (C) ultrasound‐assisted extraction.

In solid–liquid extraction, ILs enhance interactions due to their ionic nature, robustly interacting with matrices to increase permeability and extraction efficiency. Their nonflammable and recyclable properties underline their sustainability as solvents, promising advancements in analytical methods and the extraction of active compounds from complex materials. The ability to tailor ILs further augments their potential for diverse applications across scientific and industrial domains [88]. Figure 6 illustrates three different methods of extraction using ILs: (A) aqueous solution extraction; (B) ATPSs; (C) UAE.

In this sense, after carefully reviewing publications, we found that 24 alkaloids were extracted, isolated, and identified using ILs as solvents (Table 3). Before extraction, comparative studies were conducted using different cations and anions to determine the most efficient solvent composition. In addition, the concentration of these ILs was evaluated, revealing that both lower and higher concentrations are less effective. Consequently, intermediate concentrations are typically favored. Furthermore, it was concluded that the structure of ILs significantly influences their physicochemical properties, thereby impacting the efficiency of alkaloid extractions.

**TABLE 3:** Summary of study conditions for alkaloid extraction using ionic liquids.

Target alkaloid(s)	Plant source	Assistance technique	IL Composition	Optimized conditions	References
Berberine (**53**), jatrorrhizine (**54**), palmatine (**55**)	*Phellodendron amurense*	Ultrasound‐assisted extraction	[Bmim][Br]	100 W ultrasonic power, 75 min, solid–liquid ratio 1:14	Wang et al. [90]
*S*‐(+)‐glaucine (**48**)	*Glaucium flavum*	Aqueous solution extraction	[C_4_C_1_im][Ace]	1 M IL‐aqueous solution, solid–liquid ratio 1:40, methanol comparison	Bogdanov et al. [91]; Keremedchieva et al. [92]
Galanthamine (**39**)	*Leucojum aestivum*	Infrared‐assisted extraction	[C_4_C_1_im]Cl	Optimized time and temperature	Svinyarov et al. [88]
Benzoylmesaconine (**62**), benzoylaconine (**63**), benzoylhypaconine (**64**), mesaconitine (**65**), hypaconitine (**66**), aconitine (**67**)	*Aconitum carmichaeli*	Aqueous two‐phase systems	[C_6_MIM]Br‐K_2_HPO_4_	[C*_n_* MIM]Br (*n* = 4, 6, 8)‐K2HPO4, varying IL alkyl chain lengths, phase separation optimization	Wang et al. [93]
Lycorine (**56**), lycoramine (**40**), galanthamine (**39**)	*Lycoris radiata*	Infrared‐assisted extraction	[C_4_MIM]Cl	0.5 M [C_4_MIM]Cl, 60% methanol, liquid‐to‐solid ratio 50:1, 10 min	Xu et al. [94]
Protopine (**68**), allocryptopine (**69**), sanguinarine (**70**), chelerythrine (**71**), dihydrochelerythrin, dihydrosanguinarine	*Macleaya cordata*	Not specified	[C_6_MIM][Br]	100 µL IL, pH 4.0, 15 min UAE, 80°C	Li et al. [89]
Vincamine (**57**), vindoline (**58**), catharanthine (**59**)	*Vinca minor*, *Catharanthus roseus*	Not specified	[BMIm]Cl	Ultrasonic bath 50°C for 30 min	Koel et al. [95]
Berberine (**53**), palmatine (**55**), jatrorrhizine (**54**), magnoflorine (**60**), phellodendrine (**61**)	*P. amurense*	Ultrasound‐assisted extraction	[C_4_mim][OAc]/DMSO	On‐aqueous solvent system with DMSO, optimized viscosity, enhanced extraction yield	Peng et al. [96]

In 2015, the use of ILs for alkaloid extraction saw notable advancements after three key studies. Wang et al. developed an extraction method for berberine (**53**), jatrorrhizine (**54**), and palmatine (**55**) from *Phellodendron amurense* Rupr. [90]. They systematically optimized extraction parameters, including varying IL compositions (different cations and anions), IL concentration, solid–liquid ratio, ultrasound power, and extraction time. Optimal conditions using [Bmim][Br] with 100 W ultrasonic power, 75 min extraction time, and a 1:14 solvent to raw material ratio yielded a high extraction efficiency of 106.7% (Table 3). Simultaneously, Bogdanov et al. and Keremedchieva et al. explored IL‐based extraction systems form (**48**) from *Glaucium flavum* Cr. [91, 92]. Bogdanov et al. compared the efficiency of 1 M [C_4_C_1_im][Ace]‐aqueous solution versus methanol, highlighting the superior performance of the IL‐based system with an optimal solid–liquid ratio of 1:40 (wt/v) [91]. Keremedchieva et al. investigated the back‐extraction capabilities of various organic solvents and found that chloroform achieved quantitative recovery of (**48**) in a single step, resulting in product purities exceeding 95%. These first studies were important milestones because they demonstrated the effectiveness of ILs in improving both the efficiency and purity of plant‐derived alkaloids extraction [92].

Shortly thereafter, Svinyarov et al. explored aqueous solutions of various hydrophilic imidazolium‐, pyrrolidinium‐, and ammonium‐based ILs as extractants to efficiently obtain (**39**) from the aerial parts of *Leucojum aestivum* L. [88]. They explored several cations and anions, optimizing conditions like extraction time, temperature, and solvent concentration. The study established optimal parameters for quantitative extraction of (**39**), leading to a method using [C_4_C_1_im]Cl for preliminary extraction followed by high‐performance liquid chromatography (HPLC) quantification in plant material. In a study focused on extracting aconitum alkaloids from *Aconitum carmichaeli* Debx. using IL‐based ATPSs (IL‐ATPS), various parameters were optimized and compared with conventional techniques. Several ILs and inorganic salts were combined, and the findings indicated that [C*_n_* MIM]Br (*n* = 4, 6, 8)‐K_2_HPO_4_ systems formed clear and stable two‐phase systems, an ideal condition for extracting those alkaloids due to effective phase separation and suitable pH conditions [93]. Notably, extraction yields increased with the alkyl chain length of the ILs from butyl to hexyl.

The application of ILs in infrared‐assisted extraction (IRAE) of alkaloids from *Lycoris radiata* was conducted [94]. Parameters of the system IL‐IRAE were systematically optimized using ILs with different cations and anions, focusing on achieving high extraction yields of (**39**), (**40**), and lycorine (**56**). Best conditions included 0.5 M [C_4_MIM]Cl combined with 60% methanol, a liquid‐to‐solid ratio of 50:1 (mL/g), an extraction time of 10 min, and a 275‐W lamp. Similarly, Li et al. (2017) developed a green extraction method for alkaloids from *Macleaya cordata*, particularly focusing on 1‐hexyl‐3‐methylimidazolium ILs with various anions (Cl^−^, Br^−^, ${\mathrm{BF}}_{4}^{-}$, ${\mathrm{PF}}_{6}^{-}$). Most water‐miscible ILs were efficient for the procedure, with [C_6_MIM][Br] showing optimal results. The study showed that extraction was influenced by the anion type and the length of the alkyl chain in the IL cation. The extraction procedure was improved by dissolving varying amounts of [C_6_MIM][Br] in water, determining that 100 µL provided the highest recovery of target alkaloids [89].

Koel et al. evaluated five imidazolium‐based ILs and nine DESs for extracting indole alkaloids from the leaves of *Vinca minor* and *C. roseus*. They focused on extracting three specific alkaloids: vincamine (**57**) from *V. minor*, and vindoline (**58**) and catharanthine (**59**) from *C. roseus*. The efficiency of ILs and DESs varied depending on the plant and compound, but both showed potential for comparable or better yields than methanol. The IL [BMIm]Cl produced extraction yields similar to methanol, especially for *C. roseus*. Extraction yields were consistent, with standard deviations between 4% and 7%. Yields for (**59**) ranged from 60.2 to 329.9 µg/g, for (**58**) from 114.8 to 659.7 µg/g in *C. roseus*, and for (**57**) from 6.0 to 173.9 µg/g in *V. minor*. The study demonstrated the potential of ILs and DESs as effective solvents for alkaloid extraction, with specific selectivity for different compounds [95].

In a recent study, Peng et al. explored using ILs for dissolving lignocellulose, selecting a 1‐butyl‐3‐methylimidazolium acetate/dimethyl sulfoxide ([C_4_mim][OAc]/DMSO) nonaqueous solvent system as a green solvent for alkaloid extraction. This system effectively extracted (**53**), (**54**), (**55**), magnoflorine (**60**), and phellodendrine (**61**) from 0.1 g of *P. amurense*. The addition of DMSO reduced the viscosity of the IL and aided in dissolving cell walls. Compared to traditional methods like heating reflux extraction and maceration extraction, incorporating UAE technology increased the yield and quality of alkaloid active substances while reducing extraction time [96].

ILs have shown significant potential since their introduction for extracting alkaloids from plant materials, achieving high efficiencies while prioritizing environmental sustainability. Their versatility, customizable through variations in both cationic and anionic components, enhances their utility and allows for tailored optimization in specific applications. Table 3 provides a comprehensive summary of ILs used in referenced studies, detailing extracted alkaloids and their respective plant sources. Moreover, the literature suggests a shift from ILs to DESs in extraction methods, highlighting the considerable promise of both solvent systems. Thus, in the next topic, we will explore advancements in plant‐derived alkaloids using DESs.

### 3.5 DES Plant Alkaloid Extraction: DES and NADES

DESs and NADESs have emerged as green alternatives to conventional organic solvents for extraction processes. DESs are a class of liquids formed by mixing two or more solid components, typically prepared under agitation and maintained at 40°C–90°C. These components include a hydrogen bond acceptor (HBA) and a hydrogen bond donor (HBD), which, when combined, exhibit unique physicochemical properties such as higher biodegradability, thermal stability, and lower toxicity [97, 98].

**FIGURE 7:**
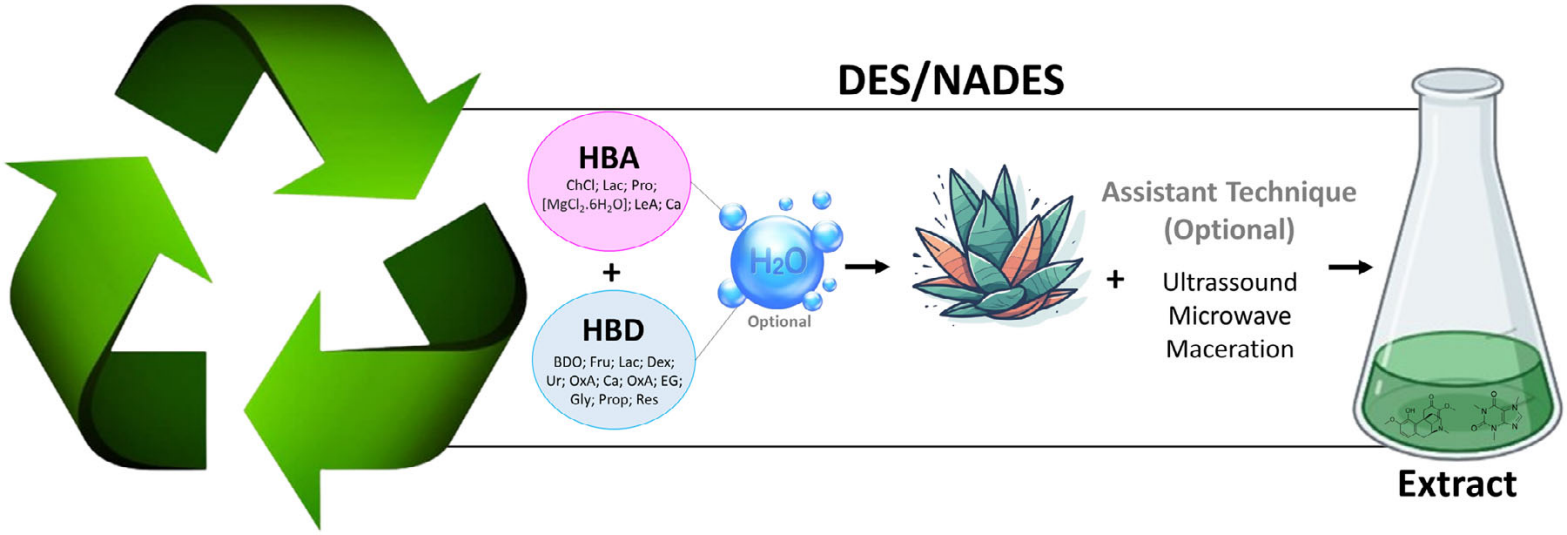
Illustration of a schematic representation of the DES/NADES extraction process. Choline chloride (ChCl); 1,4‐butanediol (BDO); fructose (Fru); lactic acid (Lac); dextrose (Dex); water (H_2_O); citric acid (Ca); l‐proline (Pro); ethylene glycol (EG); magnesium chloride hexahydrate ([MgCl_2_·6H_2_O]); urea (Ur); glycerol (Gly); propanediol (Prop); levulinic acid (LeA); resorcinol (Res); oxalic acid (OxA).

Common HBAs used in the preparation of DESs include choline chloride and l‐proline. These are combined in different molar ratios with HBDs, such as carboxylic acids, polyols, and alcohols. The choice of HBA and HBD components, as well as their molar ratios, can lead to more selective and specific extraction processes, as illustrated by Takla et al. [99]. Their study demonstrates how varying the combination of HBA and HBD (Figure 7) can effectively extract groups of alkaloids or specific alkaloids, highlighting the flexibility and efficiency of using DESs for extractions [100, 101].

In addition, NADES are a subclass of DESs, consisting mainly of natural compounds such as sugars and amino acids. This subclassification is inspired by empirical observations that plants can use eutectic mixtures to survive under extreme conditions. Furthermore, the use of DES/NADES is often assisted by complementary techniques such as maceration, MAE and UAE to optimize the transfer of target compounds from the matrix to the solvent and to reduce the extraction time [102]. Scientific studies underscore the benefits of using DES and NADES over conventional solvents. These advantages include ease of preparation, targeted extraction capability through specific combinations of HBAs and HBDs, the ability to simultaneously extract both polar and nonpolar compounds, low cost of raw materials, and improved sustainability of the extraction methods [103].

In contrast to the benefits of using DES/NADES in alkaloid extractions, several challenges deserve attention. Chief among them is the high viscosity of these mixtures, which complicates their analysis using HPLC and limits their suitability for gas chromatography due to their low volatility. Moreover, the high viscosity can impede the efficiency of the extraction process. Other critical factors include temperature, solid/liquid ratio, and extraction time. Of particular concern is the water content in these mixtures, which, while potentially reducing viscosity, can also excessively increase polarity and interfere with hydrogen bonding interactions between HBAs and HBDs, thereby adversely affecting extraction efficiency.

The landmark study performed by Wang et al. introduced the use of DES for alkaloid extraction from *Camellia sinensis* [103]. They explored combinations of choline chloride with other components such as 1,4‐butanediol, ethylene glycol, various butanediols, hexanediol, and acids like malonic, lactic, and malic, as well as acetamide, methylurea, and urea. The primary alkaloids extracted were (**5**) and (**51**), with the combination yielding the highest amounts of these compounds identified as choline chloride: 1,4‐butanediol (1:3) with 50% water (Table 4). Moreover, the study highlighted that adding water in appropriate proportions to eutectic mixtures significantly mitigated the challenge of high viscosity, thereby improving extraction performance. Optimal yields for (**5**) and (**51**) were achieved when DESs were blended with approximately 40−60 wt% of water. In addition, the study compared various DES‐assisted extraction techniques, including mechanical extraction, ultrasound, and microwave methods, with mechanical extraction proving particularly efficient in extracting the target compounds [104].

**TABLE 4:** Summary of study conditions for alkaloid extraction using supercritical fluid extraction.

Target alkaloid(s)	Plant Source	Part	Extraction for	Solvent composition	Assistance technique	References
Caffeine (**5**) (22.64 mg/g) and theobromine (**51**) (0.3573 mg/g)	*Camellia sinensis*	Leaves	DES	ChCl:BDO (1:3) with 50% H_2_O	Mechanochemical extraction, ultrasound, microwave	Wang et al. [103]
Lycorine (**56**) (1.26 mg/mL), crinine (**74**) (0.68 mg/mL) crinamine (**76**) (0.69 mg/mL)	*Crinum bulbispermum*	Bulbs	NADES	ChCl:Fru (5:2) with 20.57% H_2_O	Ultrasound	Shawky et al. [105]
Lycorine (**56**) (0.845 mg/mL), crinine (**74**) (0.563 mg/mL), and crinamine (**76**) (0.483 mg/mL)	*Crinum powellii*	Bulbs	NADES	ChCl:Fru (5:2) with 35% H_2_O	Ultrasound	Takla et al. [99]
Sinomenine (**77**) (2 g/100 g); magnoflorine (**60**) (1.5 g/100 g); berberine (**53**) (8 g/100 g); rutaecarpine (**78**) (0.06 g/100 g); evodiamine (**79**) (0.01 g/100 g); tetrandrine (**80**) (0.7 g/100 g); fangchinoline (**81**) (1.2 g/100 g); matrine (**82**) (0.01 g/100 g); ammothamnine (**83**) (2.6 g/100 g); epiberberine (**84**) (1.5 g/100 g); palmatine (**55**) (2 g/100 g); coptisine (**85**) (2.8 g/100 g)	*Caulis sinomenii*, *Coptis chinensis*, *Stephania tetrandra*, *Tetradium ruticarpum*, and *Sophora flavescens*	Plant material	DES	ChCl:Lac (1:2) with 30% H_2_O	Ultrasound	Jiang et al. [101]
Harmaline (**19**) (nd), piperine (**86**) for *Larrea cuneifolia* (33.13 ± 0.39 µg/mL), (**86**) for *L. divaricata* (44.84 ± 0.46 µg/mL); caffeine (**5**) (nd)	*L. cuneifolia*, *Larrea divaricata*, *Thymus vulgaris*, and *Origanum vulgare*	Plant material	NADES	Lac:Dex (5:1) with 15% H_2_O	Ultrasound	Espino et al. [106]
Caffeine (**5**) (CP 0.55 g/100 g; CH, reaching 0.10/100 g), theobromine (**51**) CH 0.55 g/100 g)	*Coffee*, *cocoa*, *pod husk*	Coffee pulp (CP), cocoa husk (CH), and pod husk (CPH)	DES	ChCl:Lac:H_2_O (1:2:1.5)	Heat stirring‐assisted extraction (HSE) or ultrasound‐probe assisted extraction (UPAE)	Ruesgas‐Ramon et al. [107]
Catharanthine (**59**) (60.2–329.9 µg/g), vincamine (**57**) (114.8–659.7 µg/g), vindoline (**58**) (6.0 and 173.9 µg/g)	*Catharanthus roseus* and *Vinca minor*	Leaves	DES	ChCl:Ur (1:1 and 1:2) with 25% H_2_O	Ultrasound	Koel et al. [95]
Caffeine (**5**) (1.599 mg/g) and theobromine (**51**) (5.004 mg/g)	*Cocoa bean shell (CBS)*	Husk	DES	ChCl:BDO (1:2) with 30% H_2_O	Ultrasound	Pavlović et al. [108]
Boldine (**87**) (2.36 mg/g)	*Peumus boldus*	Leaves	NADES	Pro:OxA (1:1) with 20% H_2_O	Ultrasound	Torres‐Vega et al. [110]
Berberine (**53**) (10.82 mg/g) and palmatine (**55**) (4.74 mg/g)	*Phellodendri amurensis*	Plant material	DES	ChCl:Ca (1:2) with 30% H_2_O	Ultrasound	Li et al. [109]
Arecoline (**88**) (34.07–35.86 mg/g), arecaine (**89**) (2.11–2.25 mg/g), guvacoline (**90**) (11.12–15.89 µg/g), and guvacine (**91**) (0.53–0.85 µg/g)	*Areca catechu* L.	Seeds	DES	Pro:EG (1:2) and Pro:Lac (1:2)	Ultrasound	Wang et al. [111]
Betalains (betacyanin and betaxanthins) (3.65–3.99 mg/g)	*Beta vulgaris*	Plant material	DES	[MgCl_2_·6H_2_O]:Ur (2:1) and (1:1)	Ultrasound	Hernández‐Aguirre et al. [112]
Protopine (**68**) (0.49 mg/g), chelidonine (**44**) (2.80 mg/g), berberine (**53**) (0.33 mg/g), chelerythrine (**71**) (2.31 mg/g), coptisine (**85**) (1.07 mg/g), and sanguinarine (**70**) (1.71 mg/g)	*Chelidonium majus*	Roots and shoots	NADES	MT, MC, and TC in different molar ratio	Ultrasound	Strzemski et al. [116]
*O*‐Nornuciferine (**92**) (0.069 g/100 g), *N*‐nornuciferine (**93**) (0.152 g/100 g), nuciferine (**94**) (0.334 g/100 g), and roemerine (**95**) (0.041 g/100 g)	*Nelumbo nucifera*	Leaves	DES	ChCl:Prop (1:4) with 30% H_2_O	Ultrasound	Liu et al. [113]
Skimmianine (**96**) (76.6 µg/g), dictamnine (**97**) (97.85 µg/g), and evodiamine (**79**) (531.67 µg/g)	*Evodia lepta*	Roots	DES	LeA:Gly (1.5:1) containing 30% K_2_HPO_4_	Microwave with the exception of extractions with methanol mixtures, which were carried out at room temperature	Yu et al. [115]
Fangchinoline (**81**) (7.23 mg/g) and tetrandrine (**80**) (13.36 mg/g)	*Stephania tetrandra*	Roots	DES	ChCl:EG (1:2) with 20% H_2_O	Ultrasound	He et al. [54]
No specification, alkaloid total apomorphine derivatives (392.3 ± 8.0 µg ACE/mL) (ACE: apomorphine hydrochloride equivalent)	*Nicotiana tabacum*	Inflorescences	NADES	ChCl:Ur:H_2_O (1:2:1.5)	Maceration	Leal et al. [116]
Atisinium (**99**) (Method 1: 85.73 ± 4.48 mg/g), lappaconitine (**100**) (Method 1: 7.99 ± 0.11 mg/g), 2‐*O*‐cinnamoyl hetisine (**101**) (Method 2: 11.67 ± 0.15 mg/g)	*Aconitum heterophyllum*	Roots	NADES	Method 1—Lac:Gly (1:1) and Method 2—ChCl:Res (1:1)	Ultrasound	Sharma et al. [118]
No specification, alkaloid total apomorphine derivatives (1123.0 ± 7.0 µg ACE/mL) (ACE: apomorphine hydrochloride equivalent)	*N. tabacum*	Leaves	NADES	ChCl:Ur:H_2_O (1:2:1.5)	Maceration	Leal et al. [117]
Caffeine (**5**) (21.61 ± 0.95 mg/g) and theobromine (**51**) (2.02 ± 0.10 mg/g)	*Ilex paraguariensis*	Plant material	NADES	Ca:Gly:H_2_O (1:3:9)	Ultrasound	Cunha et al. [104]

*Note*: Choline chloride (ChCl); 1,4‐butanediol (BDO); fructose (Fru); lactic acid (Lac); dextrose (Dex); water (H_2_O); citric acid (Ca); l‐proline (Pro); ethylene glycol (EG); magnesium chloride hexahydrate ([MgCl_2_·6H_2_O]); urea (Ur); glycerol (Gly); menthol–thymol (MT); menthol–camphor (MC); thymol–camphor (TC); propanediol (Prop); levulinic acid (LeA); resorcinol (Res); oxalic acid (OxA). Other combinations were tested, but only the most important of them are in this study.

In 2018, a noticeable trend toward the increased use of NADES over DES emerged. Two studies published that year focused on these solvents: one examining both types and another specifically exploring NADES. Both studies investigated the extraction of alkaloids such as (**56**), crinine (**74**), and crinamidine (**75**) from the plant species *Crinum bulbispermum* and *C. powellii* [99, 105].

Shawky et al. used DES/NADES consisting of choline chloride and fructose in a 5:2 molar ratio, with varying water concentrations (7%–20.57% v/v), and ultrasound assistance to extract alkaloids from *C. bulbispermum* bulbs. They obtained concentrations of (**56**) (1.26 mg/mL), (**74**) (0.68 mg/mL), and crinamine (**76**) (0.69 mg/mL) [105]. Similarly, Takla et al. employed NADES with the same choline chloride and fructose molar ratio, but with 35% water and UAE to obtain alkaloids from *C. powellii* bulbs. They achieved concentrations of (**56**) (0.845 mg/mL), (**74**) (0.563 mg/mL), and (**76**) (0.483 mg/mL) [99]. These studies demonstrate the effectiveness of DES and NADES in extracting alkaloids, highlighting the role of water content and ultrasound in enhancing extraction efficiency. The consistent use of the choline chloride‐fructose system across both studies underscores its applicability and effectiveness in alkaloid extraction from different plant species.

In 2019, Jiang et al. employed DESs to extract alkaloids from several medicinal plants, including *Caulis sinomenii*, *Coptis chinensis*, *Stephania tetrandra*, *Tetradium ruticarpum*, and *S. flavescens*. They tested 75 different mixtures with varying molar ratios. Focusing on *C. sinomenii*, the study utilized a mixture of choline chloride and lactic acid (1:2), supplemented with 30% water and assisted by ultrasound. The extraction yielded the following alkaloids: Sinomenine (**77**) (2.0 g/100 g), (**60**) (1.5 g/100 g), (**53**) (8.0 g/100 g), rutaecarpine (**78**) (0.06 g/100 g), evodiamine (**79**) (0.01 g/100 g), tetrandrine (**80**) (0.7 g/100 g), fangchinoline (**81**) (1.2 g/100 g), matrine (**82**) (0.01 g/100 g), ammothamnine (**83**) (2.6 g/100 g), epiberberine (**84**) (1.5 g/100 g), (**55**) (2.0 g/100 g), and coptisine (**85**) (2.8 g/100 g) [101].

The extraction study of *Larrea cuneifolia* and *L. divaricata* focused on obtaining alkaloids like (**5**), (**19**), and piperine (**86**) using NADES [106]. The NADES composition used was lactic acid and dextrose (5:1), with the addition of 15% water (v/v), and ultrasound was employed to enhance extraction efficiency. Concentrations obtained for (**86**) were 33.13 ± 0.39 µg/mL in *L. cuneifolia*, and 44.84 ± 0.46 µg/mL in *L. divaricata*.

Ruesgas‐Ramon et al. applied the DES approach to extract alkaloids from coffee pulp (CP), cocoa husk (CH), and pod husk (CPH). Their DES formulations, featuring choline chloride, lactic acid, and water (1:2:1.5), were tested alongside variants containing betaine, glycerol, and 1,4‐butanediol. Comparative conventional ethanol‐based methods demonstrated slightly superior extraction efficiencies overall, with modest differences in yields across methods. The most effective combination (choline chloride acid) yielded 0.55 g/100 g of (**5**)from CP with ultrasound assistance, 0.10 g/100 g of (**5**) from CH with heat stirring‐assisted extraction, and 0.55 g/100 g of (**51**) from CH with ultrasound [107].

Pavlović et al. conducted a study on alkaloid extraction from cocoa bean shell (CBS) using a DES system composed of choline chloride and butan‐1,4‐diol (1:2) with 30% of water. The extraction process involved microwave heating, and 16 different mixtures with various molar ratios were tested. The main alkaloids extracted were (**5**) and (**51**), yielding 1.599 and 5.004 mg/g, respectively [108]. Various combinations of DESs were tested to extract alkaloids from *C. roseus* and *V. minor* leaves. These DESs included mixtures of choline chloride with urea in ratios (1:1 and 1:2), supplemented with 25% of water. In addition, other DES combinations evaluated were choline chloride:acetamide, choline chloride:urea, choline acetate:urea, choline chloride:glycerol, choline chloride:maltose, menthol:formic acid, menthol:acetic acid and menthol:lactic acid. Ultrasonic extraction was utilized to enhance efficiency. Results showed (**59**) concentrations ranging from 60.2 to 329.9 µg/g and (**58**) concentrations from 114.8 to 659.7 µg/g for *C. roseus*. (**57**) was extracted from *V. minor* with yields ranging from 6.0 to 173.9 µg/g [95].

Furthermore, the main alkaloids extracted from *Phellodendri amurensis* were (**53**) (10.82 mg/g) and (**55**) (4.74 mg/g) [109]. The study utilized a DES composed of choline chloride and citric acid (1:2 molar ratio) with 30% water for extraction, employing ultrasound as the processing method. Other combinations of choline chloride with citric acid, malic acid, levulinic acid, succinic acid, lactic acid, ethylene glycol, glycerol, sucrose, and xylitol in different molar ratios were also investigated. Torres‐Vega et al. used a NADES composed of l‐proline and oxalic acid (1:1) with 20% of water. In addition, they tested other combinations of choline chloride and l‐proline with 1,2‐propanediol, glycerol, lactic acid, levulinic acid, and citric acid in various molar ratios. The process was enhanced by UAE and MAE for the extraction of boldine (**87**) from the leaves of *Peumus boldus*, with a yield of 2.362 ± 0.055 mg/g [110].

Arecoline (**88**) (34.07–35.86 mg/g), arecaine (**89**) (2.11–2.25 mg/g), guvacoline (**90**) (11.12–15.89 µg/g), and guvacine (**91**) (0.53–0.85 µg/g) were the main alkaloids extracted from *Areca catechu* L. seeds [111]. The study utilized UAE and a DES composed of l‐proline and ethylene glycol (1:2) and l‐proline and lactic acid (1:2). However, other DES combinations were tested, varying the molar ratios (Table 4). All prepared DESs were diluted with 30% water (v/v) to optimize extraction.

Another study focused on the extraction of betalains, including betacyanins and betaxanthins, from *Beta vulgaris*. The extraction method employed was UAE using magnesium chloride hexahydrate [MgCl_2_·6H_2_O] (HBA) and urea (HBD), in two molar ratios: (2:1) and (1:1). This study aimed to investigate the efficiency of these DESs in extracting natural pigments. The results showed that betalains were extracted with yields ranging from 3.65 to 3.99 mg/g, depending on the molar ratio used [112].

Another study using DES in 2022 was developed by Liu et al. [113], who focused on the extraction of *O*‐nornuciferine (**92**), *N*‐nornuciferine (**93**), nuciferine (**94**), and roemerine (**95**) from lotus leaves (*Nelumbo nucifera*). Seventy different mixtures with varying molar ratios were tested using ultrasound assistance. The results showed extraction yields of 0.069 g/100 g for (**92**), 0.152 g/100 g for (**93**), 0.334 g/100 g for (**94**), and 0.041 g/100 g for (**95**) under the condition of choline chloride–propanediol (1:4) containing 30% water, and a solid–liquid ratio of 1:100 g/mL.

The extraction of (**44**), (**53**), (**85**), (**68**), (**71**), and (**70**) from *C. majus* was performed using UAE. In a study by Strzemski et al., solvents such as menthol–thymol (MT), menthol–camphor (MC), and thymol–camphor (TC) were used in various molar ratios. These ratios included MT (5:5 and 6:4), MC (6:4 and 7:3), and TC (4:6 and 5:5), with acidified water and methanol serving as control extractants. The extraction results showed yields of (**68**) (0.49 mg/g), (**44**) (2.80 mg/g), (**53**) (0.33 mg/g), (**71**) (2.31 mg/g), (**85**) (1.07 mg/g), and (**70**) (1.71 mg/g) under these experimental conditions [114].

In 2023, several studies focused on the obtention of bioactive alkaloids. Yu et al. investigated the extraction of (**79**), skimmianine (**96**), and dictamnine (**97**) from *Evodia lepta* roots using levulinic acid and glycerol (1.5:1) with 30% of K_2_HPO_4_ solution, achieving yields of 76.60 µg/g for (**96**), 97.85 µg/g for (**97**), and 531.67 µg/g for (**79**) under MAE conditions [115]. Concurrently, He et al. explored DES for extracting (**81**) and (**80**) from *S. tetrandra* roots, utilizing choline chloride‐glycol (1:2) with 20% water, and achieved yields of 7.23 mg/g for (**81**) and 13.36 mg/g for (**80**) using UAE [54]. In addition, Leal et al. [116, 117] investigated NADES for extracting alkaloids from *Nicotiana tabacum*, obtaining 392.3 ± 8.0 µg ACE/mL of apomorphine hydrochloride (**98**) from inflorescences [117] and 1123.0 ± 7.0 µg ACE/mL from leaves [117] using choline chloride:urea:water (1:2:1.5). Sharma et al. also utilized NADES for extracting atisinium (**99**), lappaconitine (**100**), and 2‐*O*‐cinnamoyl hetisine (**101**) from *Aconitum heterophyllum* roots, achieving yields of 85.73 ± 4.48, 7.99 ± 0.11, and 11.67 ± 0.15 mg/g, respectively, under various DES compositions [118]. In addition, Fernández et al. investigated NADES for extracting (**5**) and (**51**) from *Ilex paraguariensis* using CGH (citric acid:glycerol:water), LGH (lactic acid:glucose:water), and ChGH (choline chloride:glycerol:water), yielding 21.61 ± 0.95 mg/g of (**5**) and 2.02 ± 0.10 mg/g of (**51**) under UAE conditions [119].

DESs have evolved significantly for alkaloid extraction from various plants. Techniques like ultrasound, mechanical extraction, and microwave assistance have further improved yields, with mechanical methods standing out for their robustness in extracting alkaloids. These developments underscore the ongoing innovation in solvent design and extraction techniques, paving the way for enhanced bioactive compound recovery and broader applications in pharmaceutical and agricultural industries.

#### 3.5.1 Most Commonly Conditions Used in DES/NADES Systems

**FIGURE 8:**
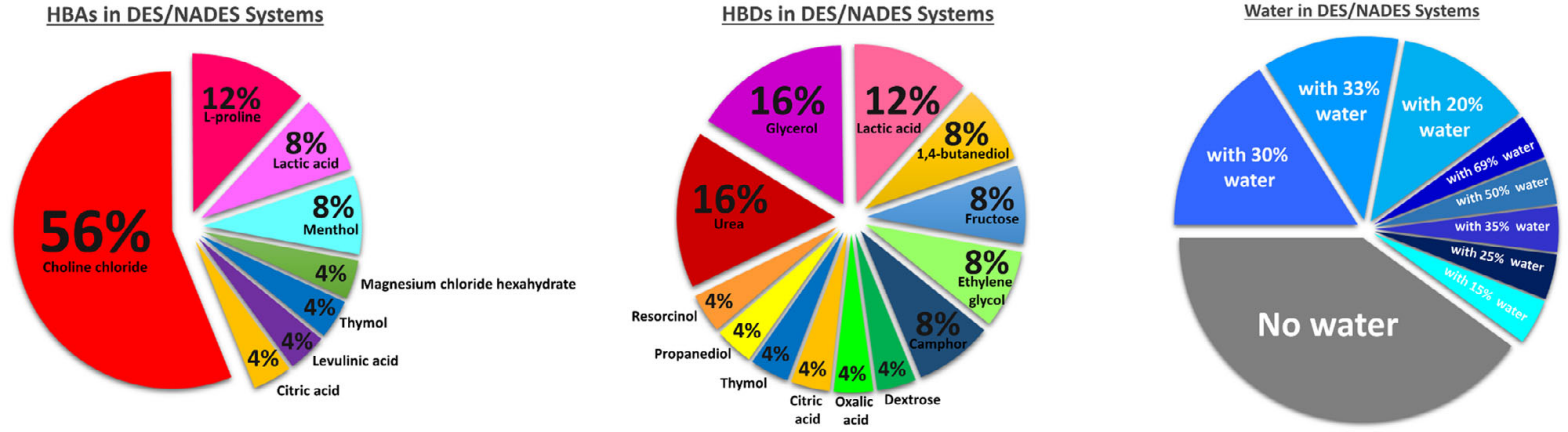
The most commonly employed HBA, HBD, and water conditions in alkaloid extraction using DES/NADES from 2014 to 2023.

In the selected studies, various combinations of HBA and HBD were used with variations in molar ratios. In addition, in most cases, water was added to the system to reduce viscosity. In this context, as illustrated in Figure 8, it can be observed that in terms of HBA, the most used compounds are choline chloride, present in 56% of the studies, followed by l‐proline in 12%, and lactic acid and menthol, both with 8%. Other compounds include magnesium chloride hexahydrate, thymol, levulinic acid, and citric acid, each representing 6% of the studies.

Among the most frequently used compounds, such as HBD, urea and glycerol stand out, each appearing in 16% of the studies, followed by lactic acid in 12%. Compounds such as 1,4‐butanediol, fructose, ethylene glycol, and camphor were used in 8% of the studies. Other compounds, including dextrose, oxalic acid, citric acid, thymol, propanediol, and resorcinol, were utilized in 4% of the studies.

Regarding the addition of water to the DES/NADES systems, 40% of the studies did not incorporate water into the eutectic solvent system. In the remaining studies, the proportion of added water varied between 15% and 69%. In 16% of the studies, 30% water was added; 12% of the studies used 20% water, and another 12% employed 33%. The remaining proportions include 69% of water (4% of the studies), 50% of water (4%), 35% of water (4%), 25% of water (4%), and 15% of water (4%).

Thus, it becomes evident that choline chloride is the most widely used component as an HBA, standing out in a significant number of studies. Similarly, urea and glycerol emerge as the most utilized HBDs, indicating their relevance in the processes analyzed. Furthermore, it can be observed that in the majority of studies using these compounds, there is a common practice of adding water to the system, with variations in the proportions employed. However, it is important to note that a considerable portion of the authors (40% of the studies) chose not to include water in the DES/NADES systems, preferring to maintain the system in its original eutectic form without dilution.

The inclusion of water in eutectic systems is influenced by various factors, including the desired properties, solubility, and stability of the target compounds. Adding water is often beneficial, particularly for extracting NPs, as it significantly improves extraction efficiency for polar substances. This is evident in the data from this survey, where 60% of studies utilized water in their systems. Water's ability to lower the viscosity of DES/NADES mixtures enhances their manageability and increases the solubility of hydrophilic compounds, aiding in the extraction process. However, water's presence is not always advantageous. It can negatively impact compounds prone to hydrolysis and, in some organic synthesis or separation processes, might affect the selectivity or efficiency of the procedure. Therefore, the decision to add water must be carefully considered, balancing the benefits of enhanced extraction efficiency and reduced viscosity against the potential downsides related to compound stability and process selectivity [120, 121, 122].

For a more comprehensive view, Table 5 correlates the advantages, disadvantages, main components of each solvent, and the most common uses of each complementary extraction technique.

**TABLE 5:** Comparison of advantages, disadvantages, composition of mixtures, and main uses of nonconventional and conventional solvents.

	MAE/UAE	IL	SFE	DES	NADES	Conventional solvents
Advantages	Improve extraction efficiency, speed, and cost‐effectiveness while providing sustainable, high‐purity extracts with reduced solvent and energy consumption	Negligible vapor pressure, nonflammability, high thermal stability, low chemical reactivity, excellent solubility, extraction efficiency, a broad liquidus range, enhances interactions with solid matrices, increasing permeability, and reducing solvent consumption	Uses supercritical CO₂ for selective, efficient extraction while reducing organic solvent use	Ease of preparation, targeted extraction with HBA and HBD, capability to extract polar and nonpolar compounds simultaneously, low cost of raw materials, sustainability	Greater biocompatibility, environmental sustainability, low toxicity, renewable sources	Ease of preparation, targeted extraction with HBA and HBD, capability to extract polar and nonpolar compounds simultaneously, low cost of raw materials, sustainability
Disadvantages	Requires precise optimization and longer extraction times to prevent degradation	Concentration and IL structure influence	Requires precise optimization of parameters to achieve good yields and maintain compound stability	Variable biocompatibility potential depending on chosen components, high viscosity	Greater difficulty in preparing a stable eutectic mixture from natural compounds, high viscosity	High environmental toxicity
Main components of mixtures	Not applicable	Organic cations paired with inorganic or organic anions	CO_2_ as solvent	HBA and HBD, often synthetic compounds	Sugars, amino acids, organic acids	Derivatives of hydrocarbons and other synthetic compounds
Main uses	Targeted heating of specific metabolites in plant samples for more efficient extraction	Widely used in synthesis, catalysis, electrochemistry, and analytical chemistry	Ideal for pharmaceutical and research applications	Extraction of bioactive compounds, catalysis, materials synthesis	Extraction of natural products, biocatalysis, food preservation	Chemical and pharmaceutical industries, large‐scale extraction operations

## 4 Conclusion and Perspectives

Modern techniques, such as SFE, MAE, and UAE, have revolutionized the field of NP chemistry, especially in extracting bioactive compounds like plant‐derived alkaloids. These strategies offer substantial advantages over traditional solvent extraction methods by significantly enhancing extraction efficiency, reducing processing times, and improving the purity of extracted compounds. Despite these advancements, further research is needed to standardize and optimize extraction conditions to maximize yield and ensure reproducibility across different plant species and alkaloid classes.

Looking forward, integrating these advanced extraction techniques with sustainable solvents, such as ILs, DESs, and NADESs, aligns with the United Nations' Sustainable Development Goals (SDGs). These green solvents, derived from renewable resources, support SDG 12 (Responsible Consumption and Production) by promoting sustainable chemical use and reducing waste. They also contribute to SDG 13 (Climate Action) by lowering the carbon footprint associated with traditional organic solvents.

Future research should focus on developing novel DES and NADES formulations tailored for specific alkaloid classes and plant matrices, advancing SDG 9 (Industry, Innovation, and Infrastructure) through sustainable technology and process optimization. This approach will enhance solvation properties, improve extraction yield and purity, and minimize environmental impact.

Furthermore, integrating computational modeling and process intensification techniques can streamline extraction methods, further supporting SDG 9 and SDG 8 (Decent Work and Economic Growth) by boosting industrial efficiency and economic viability. Computational tools can predict solvent–solute interactions and guide the design of optimized extraction conditions, while process intensification improves throughput and scalability. These advancements make green extraction technologies practical for industrial‐scale applications in pharmaceuticals, cosmetics, and functional foods.

In conclusion, modern extraction techniques, coupled with sustainable solvents and advanced computational methods, represent the future of alkaloid extraction, offering efficient, eco‐friendly, and economically viable solutions for various industry segments. In addition, the integration of artificial intelligence (AI) and machine learning (ML) will enhance the optimization and scaling of alkaloid extraction, allowing for more efficient and targeted processes. These technologies will work alongside sustainable extraction methods, creating adaptive protocols that maximize yield and reduce waste. Furthermore, the adoption of circular economy principles and biorefinery concepts will enable the repurposing of waste materials from extraction, reducing environmental impact and promoting resource reuse. Together, these advancements will foster a more sustainable, efficient, and economically viable future for NP exploration, aligning with global sustainability goals.

## Author Contributions

**Thiago André Moura Veiga**: writing – original draft preparation and editing, writing – review and editing, supervision, funding acquisition. **Victor Menezes Sipoloni**: writing – original draft preparation and editing, writing – original draft preparation, writing – review and editing. **Milena Costa Bassicheto**: writing – original draft preparation. **Maria Vitória de Oliveira**: writing – original draft preparation. **Ana Beatriz dos Santos de Souza**: writing – original draft preparation. **Gabriella Melo Alves**: writing – original draft preparation.

## Conflicts of Interest

The authors declare no conflict of interest.

## Data Availability

The data that support the findings of this study are available from the corresponding author upon reasonable request.
